# Chitosan-propolis nanoparticle formulation demonstrates anti-bacterial activity against *Enterococcus faecalis* biofilms

**DOI:** 10.1371/journal.pone.0174888

**Published:** 2017-03-31

**Authors:** Teik Hwa Ong, Ebenezer Chitra, Srinivasan Ramamurthy, Rajinikanth Paruvathanahalli Siddalingam, Kah Hay Yuen, Stephen Periathamby Ambu, Fabian Davamani

**Affiliations:** 1 School of Postgraduate Studies, International Medical University, Bukit Jalil, Kuala Lumpur, Malaysia; 2 Division of Applied Biomedical Science and Biotechnology, School of Health Sciences, International Medical University, Bukit Jalil, Kuala Lumpur, Malaysia; 3 School of Pharmaceutical Sciences, Universiti Sains Malaysia, Gelugor, Penang, Malaysia; Central University of Tamil Nadu, INDIA

## Abstract

Propolis obtained from bee hives is a natural substance with antimicrobial properties. It is limited by its insolubility in aqueous solutions; hence ethanol and ethyl acetate extracts of Malaysian propolis were prepared. Both the extracts displayed antimicrobial and anti-biofilm properties against *Enterococcus faecalis*, a common bacterium associated with hospital-acquired infections. High performance liquid chromatography (HPLC) analysis of propolis revealed the presence of flavonoids like kaempferol and pinocembrin. This study investigated the role of propolis developed into nanoparticles with chitosan for its antimicrobial and anti-biofilm properties against *E*. *faecalis*. Bacteria that grow in a slimy layer of biofilm are resistant to penetration by antibacterial agents. The use of nanoparticles in medicine has received attention recently due to better bioavailability, enhanced penetrative capacity and improved efficacy. A chitosan-propolis nanoformulation was chosen based on ideal physicochemical properties such as particle size, zeta potential, polydispersity index, encapsulation efficiency and the rate of release of the active ingredients. This formulation inhibited *E*. *faecalis* biofilm formation and reduced the number of bacteria in the biofilm by ~90% at 200 μg/ml concentration. When tested on pre-formed biofilms, the formulation reduced bacterial number in the biofilm by ~40% and ~75% at 200 and 300 μg/ml, respectively. The formulation not only reduced bacterial numbers, but also physically disrupted the biofilm structure as observed by scanning electron microscopy. Treatment of biofilms with chitosan-propolis nanoparticles altered the expression of biofilm-associated genes in *E*. *faecalis*. The results of this study revealed that chitosan-propolis nanoformulation can be deemed as a potential anti-biofilm agent in resisting infections involving biofilm formation like chronic wounds and surgical site infections.

## Introduction

Nanoparticles are widely used in healthcare sector as well as industry for varied applications including antimicrobials [1]. In medicine, they are used as drug carriers encapsulating a broad range of therapeutic agents such as siRNA, drug molecules, proteins and peptides [2–4] leading to more efficient drug delivery into the target site. Nanoparticle drug delivery system offers advantages such as better bioavailability, efficacy, solubility and encapsulation of the drug compared to conventional systems [2, 3, 5, 6]. The choice of the carrier in a nanoparticle delivery system is crucial since it can affect protection, retention and bioavailability of the drug or the natural active ingredient.

Chitosan is a natural cationic polysaccharide derived by N-deacetylation of chitin. Chitosan is reported to exhibit adhesiveness, biocompatibility and biodegradability and is widely used in biomedical and pharmaceutical applications [7–10]. It is used as a carrier to deliver non-viral genes and vaccines [11–13]. Chitosan nanoparticles are well known for their controlled drug release properties [4, 10, 14, 15] and are used for both *in vitro* and *in vivo* applications. Chitosan can be used in dental application and corneal implants, based on its ability to form thin films [16–20]; this property can be exploited for various applications, in pharmaceutical industry it is used as disintegrant and tablet binder [21–23]. Its non-toxic and non-immunogenic properties make it an ideal delivery agent for topical as well as systemic applications in medicine for treatment against microbial infections.

*Enterococcus faecalis* is a major cause for hospital-acquired infections, urinary tract infections, endocarditis and endodontic infections and has a very high prevalence of multidrug-resistance [24] with intrinsic or acquired virulence traits. It is a Gram-positive commensal bacterium that lives in the gastrointestinal tracts of humans; it can survive in a wide range of temperatures from 10°C to 45°C and can adapt to acidic, alkaline, hypotonic or hypertonic environment [25]. It is capable of forming biofilms, a slimy layer enclosing the bacteria in an extracellular polymeric substances (EPS) matrix, which is a contributing factor for antibiotic resistance. Bacteria within the biofilms are highly resistant to antibiotics and immune responses when compared to planktonic (free-living) bacteria [26].

*E*. *faecalis* is known to form biofilms in in-dwelling medical devices like catheters and implants leading to persistent infections and complicating the therapeutic use of antibiotics [12]. Bacterial biofilms are a challenge to eradicate from hospital-acquired infections or in-dwelling medical devices since they are resistant to penetration by antibiotics. Biofilm formation is usually accompanied by the expression of virulence and antibiotic resistance genes as well as alteration in the surface protein profile of the resident bacteria. Altered gene signature is one of the significant factors enabling biofilm bacteria to become resistant to antibiotics and other eradication methods [27]. Development of natural products with bioactive ingredients will therefore help overcome the issue of drug resistance in bacteria.

Propolis is a brown resinous mixture collected by honey bees, composed of flavonoids, steroids, amino acids, terpenes, phenolic and aromatic compounds. Its chemical composition depends on the location, climate, time of collection and the type of bee species [28–30]. It has been used in traditional medicine dating back to the ancient Roman times, and is reported to have anti-bacterial, anti-fungal and anti-viral properties [31].

In this study, ethanol and ethyl acetate extracts of Malaysian propolis were prepared, their chemical composition was analyzed and they were evaluated for their antibacterial and anti-biofilm activity. Chitosan-propolis nanoparticles were prepared and characterized in terms of their physical properties such as particle size, polydispersity index, zeta potential, encapsulation efficiency, surface morphology and *in vitro* release profile. They were able to inhibit *in vitro* biofilm formation by *E*. *faecalis* and also alter expression of genes related to virulence and biofilm formation.

## Materials and methods

### Propolis, chemicals and reagents

Malaysian propolis was purchased directly from bee farms. (Pahang, Malaysia) Chitosan (medium molecular weight with degree of deacetylation of 85%), sodium tripolyphosphate (TPP), caffeic acid, rutin, quercetin, cinnamic acid, luteolin, kaempferol, apigenin and pinocembrin were purchased from Sigma-Aldrich (St Louis, MO, USA). All other chemicals were purchased from Merck (Darmstadt, Germany).

### Preparation of extracts of Malaysian propolis

Propolis was extracted from the bee hives free from wax. A 20% (w/v) extract of propolis was prepared using ethanol or ethyl acetate at 37°C for 48 hours under constant agitation in a rotary shaker at 200 rpm, filtered through Whatman no. 1 filter paper and concentrated using a rotary evaporator (Buchi Rotavapor R-215, Flawil, Switzerland). The extraction yield (Final weight/Initial weight x 100) was determined for ethanol (**Eth**) and for ethyl acetate (**EA**) extracts of propolis. Stock solutions of 1 mg/mL of the respective extracts were prepared and used for further experiments.

### RP-HPLC analysis and standardization of Malaysian propolis

Agilent 1260 HPLC system (Agilent Technologies, Santa Clara, CA, USA) equipped with quaternary pump (G1311C), UV-Vis detector (G1314B) and auto sampler (G1329B) was used to characterize the Eth and EA extracts of Malaysian propolis using the standard flavonoid markers (caffeic acid, rutin, quercetin, cinnamic acid, luteolin, kaempferol, apigenin and pinocembrin). Reversed phase high performance liquid chromatography (RP-HPLC) analysis was performed using Chromolith Performance RP-18e (100–4.6 mm) HPLC analytical column (Merck, Darmstadt, Germany) equipped with a guard cartridge (Merck, Darmstadt, Germany).

Each standard solution was prepared at 1 mg/mL concentration with methanol and diluted to obtain the following concentrations: 0.1 μg/mL, 0.25 μg/mL, 0.5 μg/mL, 1 μg/mL, 2 μg/mL, 4 μg/mL, 6 μg/mL, 8 μg/mL and 10 μg/mL to be injected into the HPLC system. Elution was done with a linear gradient of 0.05% phosphoric acid in water (pH 2.5) (solvent A) and methanol (solvent B) at a flow rate of 1 mL/min. The following gradient program was used: 0 min (B-20%); 5 min (B-30%); 8 min (B-40%); 12 min (B-50%); 15 min (B-75%); 18 min (B-80%); 22 min (B-90%) and 25 min (B-100%). The chromatograms were recorded at 260 nm for 25 minutes. Linearity was calculated for the concentration range of 0.1 μg/mL to 10 μg/mL. Eth and EA extracts of Malaysian propolis were dissolved in methanol (250 μg/mL), filtered through a 0.22 μm filter (Millipore, Merck, Darmstadt, Germany) and 20 μL was injected into the HPLC system for analytical evaluations. Data are representative of at least three independent experiments.

### Detection and quantification of pinocembrin in Malaysian propolis

To identify and quantify the flavonoid pinocembrin in Malaysian propolis, pinocembrin standard solution was prepared and elution was done using a linear gradient as described above. The elution was isocratic with 55% solvent B and 45% solvent A. The chromatograms were recorded at 260 nm for 10 minutes. Linearity was calculated for the concentration range of 0.25 μg/mL to 20 μg/mL. Eth and EA extracts of propolis were prepared as described above and injected into the column. A calibration curve of pinocembrin standard was plotted and used to calculate the concentration of pinocembrin present in the extracts. Data are representative of at least three independent experiments.

### Preparation of chitosan-propolis nanoparticles

Chitosan nanoparticles encapsulated with Eth extract were prepared by ionic gelation method with modification [32]. Briefly, different concentrations of chitosan solutions (0.2–0.5% w/v) were prepared in 0.1% v/v glacial acetic acid and filtered. Sodium tripolyphosphate solution (TPP) (0.2% w/v) was prepared in deionized water. Eth extract (0.4–1.6 mg/mL) was added to chitosan solution (0.2–0.5% w/v) containing 0.4% w/v of Tween 80 under constant stirring to obtain different formulations of chitosan-propolis nanoparticles (F1 to F6) (Table 1). The mixture was then sonicated for 5 minutes and TPP solution was added drop-wise under constant stirring. The ratio of chitosan:TPP solution was maintained at 2:1 throughout the experiment. The supernatant obtained was subjected to ultracentrifugation at 25000 rpm for 20 minutes to sediment the chitosan-propolis conjugated nanoparticles, which were then subjected to further characterization.

**Table 1:** Composition of chitosan-propolis nanoparticles formulations.

*Formulation*	*Chitosan (% w/v)*	*Propolis (mg/mL)*	*Tween 80 (% w/v)*
F1	0.2	1	0.4
F2	0.3	1	0.4
F3	0.5	1	0.4
F4	0.2	0.4	0.4
F5	0.2	1.6	0.4
F6	0.2	1	-

### Physical characterization of chitosan-propolis nanoparticles

#### Analysis of particle size and zeta potential

The average particle size, zeta potential and polydispersity index (PDI) of the prepared formulations were measured by dynamic light scattering analysis by photon correlation spectroscopy (Zetasizer Nano ZS, Malvern Instruments, Malvern, UK). Data are representative of at least three independent experiments.

#### Encapsulation efficiency

The encapsulation efficiency (EE) was determined by measuring the amount of free propolis present in the different formulations. The amount of propolis in each fraction was determined by HPLC method (Agilent HPLC 1260 series, Agilent Technologies, Santa Clara, CA, USA). The amount of encapsulated propolis was calculated with the formula given below.

$$Encapsulation\;efficiency\;\left(\%\right)=\left(\frac{\left(Total\;amount\;of\;propolis-Free\;propolis\right)}{\left(Total\;amount\;of\;propolis\right)}\right)\;x\;100$$

Data are representative of at least three independent experiments.

#### Transmission electron microscopy of nanoparticles

The nanoparticles were immobilized on copper grids, dried at room temperature and stained with 2% uranyl acetate. Their shape and morphology was examined by transmission electron microscopy (FEI Tecnai G2 20S Twin Transmission Electron Microscope, Hillsboro, Oregon, USA) at Universiti Teknologi MARA, Malaysia. Data are representative of at least three independent experiments.

#### *In vitro* release of propolis from chitosan-propolis nanoparticles

*In vitro* release of the active ingredients was determined by calculating the amount of propolis released from the selected formulation (F1: 0.2% chitosan, 1 mg/mL propolis and 0.4% tween 80) with ideal properties. Chitosan-propolis nanoparticles with a final concentration of 0.5 mg/mL was dialyzed with a 10 kDa molecular weight cut off in 200 mL of phosphate buffer saline (pH 7.4) at 37°C under constant stirring. The dialysis buffer was sampled periodically (1, 2, 4, 6, 8, 12, 24, 48 h) and analyzed by HPLC to determine the amount of propolis released from the formulation over time. Data are representative of at least three independent experiments.

### Anti-bacterial activity of propolis

#### Biofilm assay

A single colony of *E*. *faecalis* (ATCC 29212) was inoculated in tryptic soy broth (TSB) and cultured overnight at 37°C. Bacterial suspension of 0.5 McFarland units was prepared; 1 ml of this suspension was added to 24-well microtiter plate (Eppendorf, Hamburg, Germany) along with Eth or EA extracts of propolis or chitosan-propolis nanoparticles formulation (F1) (100, 200 or 300 μg/mL). The plate was incubated in a shaking incubator at 37°C for 24 h at 150 rpm, the planktonic bacteria were removed and the plate was washed with saline. Biofilm bacteria were dislodged by pipetting. The number of bacteria present in planktonic form as well as biofilm were enumerated by serial dilution, plating in tryptic soy agar plates incubated at 37°C overnight followed by colony count. Similar experiment was set up to compare of pinocembrin (1 μg/mL) and propolis Eth extract (200 μg/mL equivalent to 1 μg/mL of pinocembrin). Data are representative of at least three independent experiments.

#### Crystal violet staining of biofilm

Biofilms were formed in 96-well plates for 24 hours as described above, washed with saline and stained with 0.1% crystal violet for 15 minutes followed by washing with saline to remove the excess dye. 200 μl of ethanol was added into each well to release the dye taken up by the biofilm and absorbance was measured at 600 nm using a microplate reader (OpsysMR, Dynex Technologies, Chantilly, VA, USA). The difference in absorbance with or without treatment was used to calculate the percentage reduction of biofilm mass (%). A well with pain medium without bacteria was used as a blank. Data are representative of at least three independent experiments.

#### Pre-formed biofilm assay

*E*. *faecalis* suspension of 0.5 McFarland units was prepared as mentioned above; 1 ml of this suspension was added to 24-well microtiter plate and incubated at 37°C for 16 hours at 150 rpm to facilitate the formation of biofilm. After 16 hours, the planktonic bacteria were removed, the plate was washed with saline and TSB containing Eth or EA extracts of propolis or chitosan-propolis nanoparticles formulation (F1) (200, 300 or 400 μg/mL) was added to the wells and treated for 8 hours. After incubation, the planktonic bacteria were removed and the plate was washed with saline. The number of bacteria present in planktonic form as well as in the biofilm was enumerated as described above. Data are representative of at least three independent experiments.

#### Visualization of biofilm—Fluorescent microscopy

Slime production of *E*. *faecalis* was visualized by fluorescence microscope after staining the biofilm with calcofluor white for 15 minutes in the dark. Extracellular polymeric substances (EPS) of *E*. *faecalis* was observed through a Nikon Eclipse DAPI filter (excitation filter, 340–380 nm; dichroic mirror, 400 nm; barrier filter, 435–485 nm). Data are representative of at least three independent experiments.

#### Visualization of biofilm—Scanning electron microscopy

*E*. *faecalis* biofilm was grown on glass coverslips placed in 6-well plate and treated with 100 μg/mL of propolis Eth extract, EA extract or chitosan-propolis nanoparticles for 24 hours. The biofilm on the coverslips was fixed with 2.5% (v/v) glutaraldehyde and the samples were serially dehydrated, air-dried, sputter coated with gold (SC7620 Mini Sputter Coater, Quorum Technologies, UK) and viewed with a scanning electron microscope (TM3000 Tabletop Scanning Electron Microscope, Hitachi, Japan). Data are representative of at least three independent experiments.

#### Quantitative PCR analysis of biofilm-related genes expressed by *E*. *faecalis*

RNA was extracted from the bacteria in the biofilm as well as from planktonic bacteria using RNeasy mini kit (Qiagen) following the manufacturer’s protocol. RNA was converted to cDNA using Superscript Vilo master mix (Invitrogen, USA). Real-time QPCR analysis of gene expression was performed using 25 ng of cDNA and 1.25 μM of the appropriate primers using qPCRBio SYGreen master mix (PCR Biosystems, UK) and iQ5 real-time PCR detection system (Bio-Rad Laboratories, Hercules, California, USA). The genes analyzed were cytolysin genes (*cylB*, *cylL*_*L*_, *cylL*_*S*_, *cylR1*, *cylR2*, *cylM*, *cyl1*) and virulence genes (*gelE*, *ace*, *asa*, *fsrB*, *fsrC*, *ebpA*, *ebpB*, *ebpC*, *efa*, *gls24* and *bopD*). The reference gene used was 23s rRNA. The primers used and the corresponding temperatures are listed in S1 Table. Data are representative of at least three independent experiments.

### Statistical analysis

Quantitative data are represented as mean ± standard error of mean. QPCR data groups were analyzed by one way ANOVA, followed by post-hoc Tukey’s honest significant difference (HSD). Differences between treatments were considered significant at P<0.05 probability level.

## Results

### Characterization of Malaysian propolis

#### Composition of Malaysian propolis

**Fig 1:**
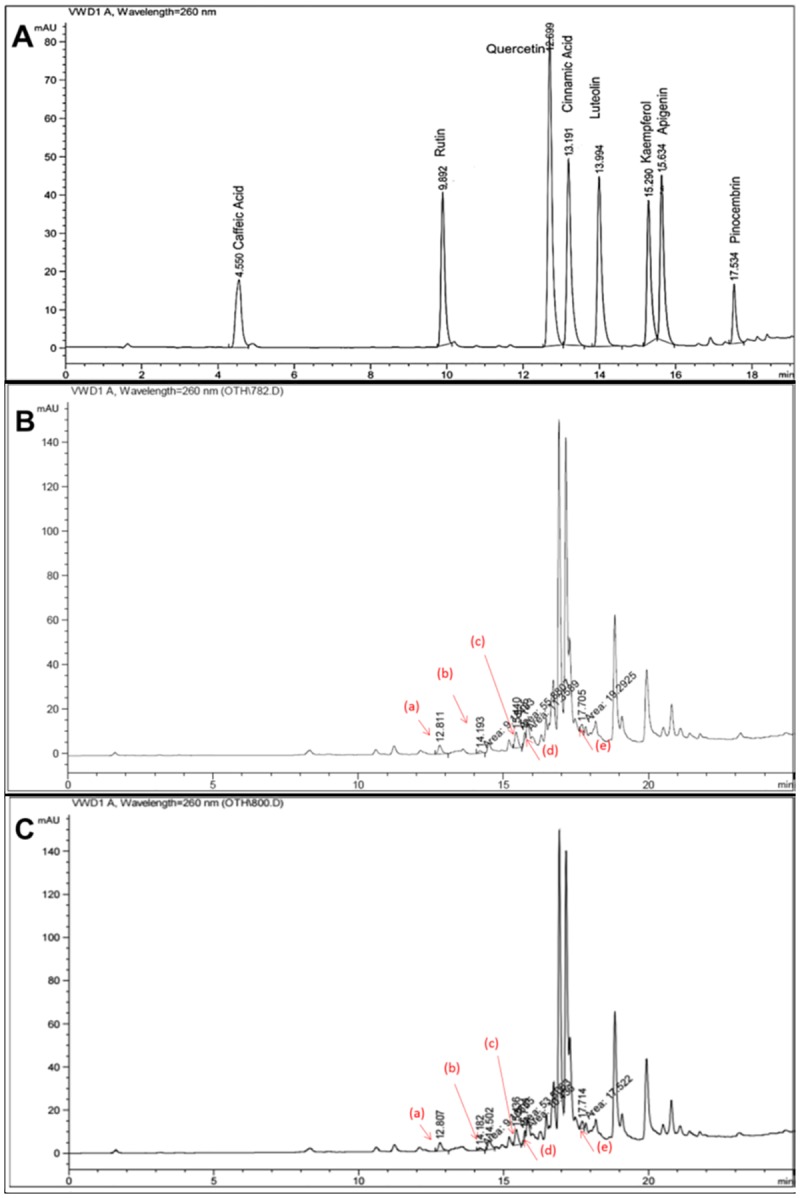
HPLC chromatograms depicting the elution profile of standard flavonoid compounds and extracts of Malaysian propolis by gradient method. A) Chromatographic profile of the flavonoid standards caffeic acid, rutin, quercetin, cinnamic acid, luteolin, kaempferol, apigenin and pinocembrin. B and C) Chromatographic profile of Malaysian propolis Eth extract (B) or EA extract (C) depicting the presence of the flavonoids (a) Quercetin (b) Luteolin (c) Kaempferol (d) Apigenin (e) Pinocembrin represented by the peaks marked with arrows. Caffeic acid, rutin and cinnamic acid were not detected in either of the extracts.

Malaysian propolis extracts (20% w/v) were prepared using ethanol (**Eth**) or ethyl acetate (**EA**) and the extraction yield obtained was 61.9% for Eth and 57.8% for EA. The extracts were analyzed by RP-HPLC using eight flavonoids (caffeic acid, rutin, quercetin, cinnamic acid, luteolin, kaempferol, apigenin and pinocembrin) as standard markers. Linearity of each standard from 0.1 μg/mL to 10 μg/mL was evaluated. The correlation coefficient observed ranged from 0.9954 to 0.9989. The representative HPLC chromatogram of the standard flavonoid markers is shown in Fig 1A. The retention time, regression equation and correlation coefficient of each standard are presented in Table 2.

**Table 2:** Calibration curve and correlation coefficients of standard flavonoids detected by HPLC.

*Compounds*	*Retention time (Minutes)*	*Regression equation*	*Correlation coefficient (r*^*2*^*)*
Caffeic acid	4.6	y = 24.086x + 0.0091	0.9975
Rutin	9.9	y = 35.999x + 5.5071	0.9989
Quercetin	12.7	y = 81.395x + 5.0073	0.9967
Cinnamic acid	13.2	y = 50.948x + 5.7558	0.9989
Luteolin	14	y = 45.031x + 2.3188	0.9989
Kaempferol	15.3	y = 37.401x + 2.9118	0.9982
Apigenin	15.7	y = 44.385x − 1.5019	0.9973
Pinocembrin	17.6	y = 11.522x + 3.3704	0.9954

RP-HPLC profile of Eth and EA extracts of Malaysian propolis is shown in Fig 1B and 1C, respectively. The flavonoids quercetin, luteolin, kaempferol, apigenin and pinocembrin were identified to be present in Malaysian propolis while caffeic acid, rutin and cinnamic acid were not detected. The concentrations of the identified flavonoids in Eth and EA extracts are presented in Table 3.

**Table 3:** Flavonoids content of Malaysian propolis.

*Standard markers*	*Malaysian propolis flavonoid content (μg/m*L*)*	
	*Ethanol extract (Eth)*	*Ethyl acetate extract (EA)*
Caffeic acid	-	-
Rutin	-	-
Quercetin	1.43	1.39
Cinnamic acid	-	-
Luteolin	0.61	0.51
Kaempferol	5.88	5.62
Apigenin	1.22	1.12
Pinocembrin	5.64	4.06

(-) indicates not present/not detectable.

The total flavonoid content was higher in Eth extract when compared to EA extract possibly due to ethanol being more polar. Among the identified flavonoids, kaempferol and pinocembrin were found to be present in significant amounts (4–5.9 μg/mL) in Malaysian propolis. Eth extract of Malaysian propolis had higher concentration of pinocembrin compared to EA extract. The concentrations of other flavonoids, quercetin, luteolin and apigenin was low (~0.5–1.5 μg/mL) and comparable between the two extracts.

#### Pinocembrin as a marker compound for Malaysian propolis

**Fig 2:**
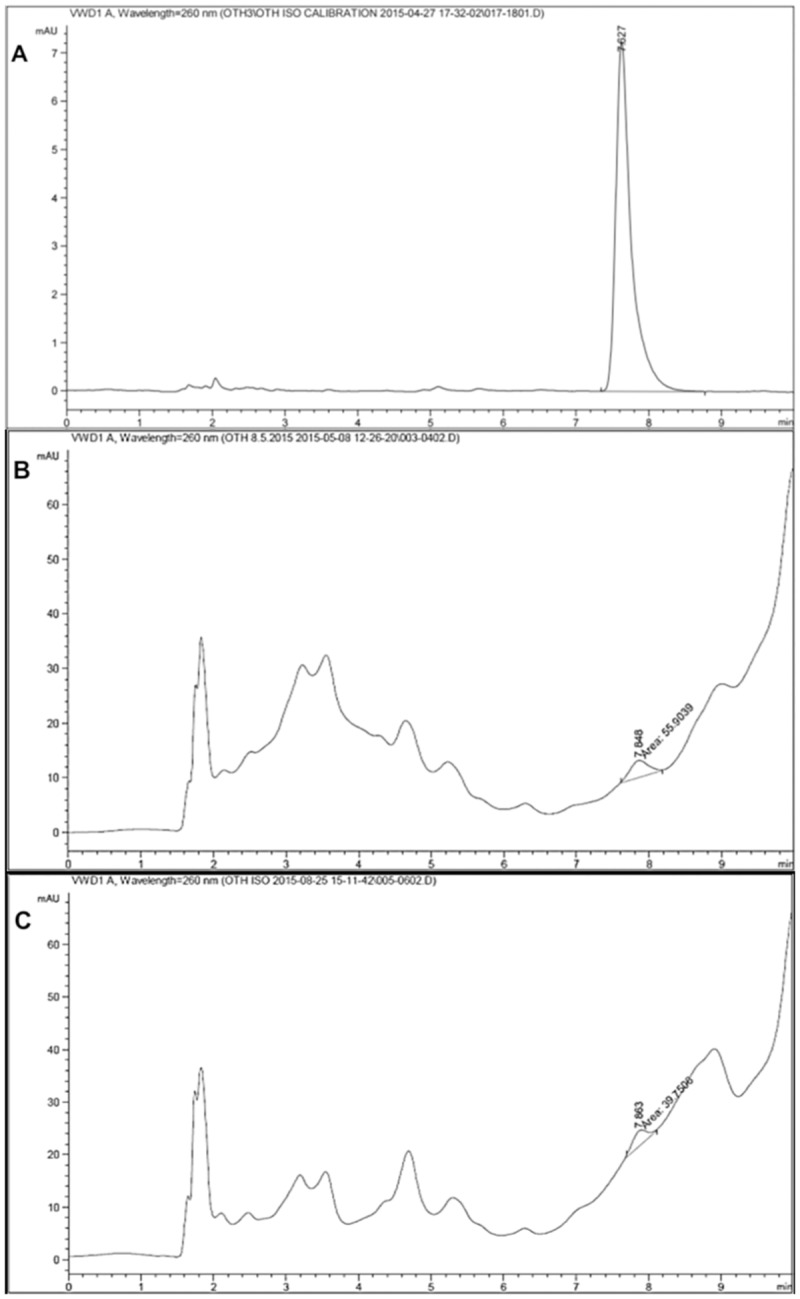
HPLC chromatograms depicting the elution profile of standard flavonoid compounds and extracts of Malaysian propolis by isocratic elution. A) Chromatograms depicting the identification and quantification of standard flavonoid marker compound pinocembrin. B and C) Chromatograms depicting the standardisation of Eth extract (B) or EA extract (C) of Malaysian propolis using pinocembrin. A comparison of the flavonoid content of Eth and EA extracts of Malaysian propolis revealed that kaempferol concentration is similar between the two extracts (Table 3), while the amount of pinocembrin present is higher in Eth extract compared to EA extract.

Malaysian propolis Eth and EA extracts were analyzed by RP-HPLC isocratic method using pinocembrin as a standard compound the respective chromatograms are illustrated in Fig 2. The calibration curve was used to determine the concentration of pinocembrin. The correlation coefficient for pinocembrin, demonstrated a good linearity ((*r*^*2*^) 0.9989) with retention time of 7.6 minutes. The concentration of pinocembrin determined by this method was as foEth extract was 5.47 μg/mL in Eth extract and 3.81 μg/mL in EA extract, respectively. Propolis Eth extract had 43% more pinocembrin than EA extract.

#### Antibacterial effect of Malaysian propolis extracts

*E*. *faecalis* was cultured in a 96-well plate to form biofilms and the ability of Eth and EA extracts of propolis in inhibiting biofilm formation was evaluated by staining the biofilms using crystal violet. Eth extract was found to inhibit bacterial biofilm formation better than EA extract as evident in S1 Fig. Therefore, Eth extract was chosen to prepare the nanoparticles due to its enhanced ability to inhibit biofilms.

### Characterization of chitosan-propolis nanoparticles

Chitosan-propolis nanoparticles were prepared as described in the methods using Eth extract of Malaysian propolis. Six different formulations (F1 to F6) were prepared by varying the concentrations of chitosan, propolis and tween 80 (Table 1). These formulations were characterized based on their average particle size, polydispersity index, zeta potential and encapsulation efficiency, the details of which are presented in Table 4.

**Table 4:** Physical characterization of the nanoparticle formulations.

*Formulation*	Average particle size (nm)*	Polydispersity index (PDI)*	Zeta potential (mV)*	*Encapsulation efficiency (%)*
Chitosan-TPP control	125.7 ± 0.53	0.438 ± 0.01	35.5 ± 0.91	-
F1	247.1 ± 1.7	0.225 ± 0.01	40 ± 0.38	88.80
F2	427.1 ± 8.9	0.499 ± 0.01	64 ± 1.89	91.43
F3	512.3 ± 15.4	0.573 ± 0.07	74.1 ± 2.75	91.11
F4	198.0 ± 3.0	0.453 ± 0.01	48.2 ± 0.85	77.65
F5	308.3 ± 6.8	0.264 ± 0.001	49 ± 1.37	88.17
F6	349.9 ± 2.3	0.371 ± 0.05	52.9 ± 3.50	88.20

*Mean ± SD.

#### Particle size and polydispersity index

A reduction in the particle size of nanoparticles is known to enhance the efficacy, solubility and bioavailability of the active drug compounds in a formulation [32]. All our formulations (F1-F6) were prepared within the concentration range of 0.2–0.5% w/v chitosan. As the concentration of chitosan in the formulation increased from 0.2% w/v to 0.5% w/v (Table 1), the average particle size of the nanoparticles increased from 247.1 nm to 512.3 nm (Table 4). This could be attributed to a combination of adsorption coagulation and bridging between chitosan and tripolyphosphate as the coating amount of chitosan increased. Decreasing the chitosan concentration below 0.2% w/v resulted in clumping of the nanoparticles while increasing chitosan concentration above 0.5% w/v resulted in formation of large, discrete, aggregated nanoparticles with particle size above 400 nm. This could be due to an increase in viscosity of the solution at high concentration and increase in particle size during emulsification.

Formulations F1 and F4, both with chitosan concentration of 0.2% were found to have an acceptable average particle size of 247 nm and 198 nm, respectively. The former was prepared with higher concentration (1 mg/mL) of propolis compared to the latter (0.4 mg/mL). The average particle size of the formulations increased with increasing concentration of propolis while addition of tween 80 reduced the average particle size of the formulation (Table 4).

Polydispersity index is an indicator of the size distribution of nanoparticles. All our formulations showed a polydispersity index ranging from 0.225 to 0.573, suggesting that they were monodispersed. Formulation F1 and F5 were found to have the lowest polydispersity index of 0.225 and 0.264, respectively. The formulation F1 with propolis concentration of 1 mg/mL was found to have lower particle size as well as low polydispersity index.

#### Zeta potential and encapsulation efficiency

Chitosan nanoparticles generally have a positive zeta potential due to the cationic properties of chitosan molecule. Positive zeta potential of chitosan enhances drug delivery by facilitating adherence to the negatively charged cell membrane [33]. Stability of the nanoparticles in a suspension is affected by their zeta potential [34]. As the magnitude of zeta potential increases, the electrostatic repulsion between the particles becomes greater leading to a more stable colloidal dispersion. Our formulations were found to have a positive zeta potential between +35.5 mV to +74.1 mV (Table 4). Formulations with chitosan concentration of 0.2% w/v (F1, F4, F5, and F6) had zeta potential in the range of +45.2 mV to +52.9 mV, while an increase in chitosan concentration increased the zeta potential to above +60 mV. In nanoparticle delivery system, the encapsulation efficiency is defined as the drug carrying capacity. All our samples had an encapsulation efficiency of 88% or above except formulation F4, which had a lower encapsulation efficiency of 77%.

Based on all the above parameters, formulation F1 (0.2% w/v chitosan and 1 mg/mL propolis) was found to be ideal with an average particle size of 247.1 nm, polydispersity index of 0.225, zeta potential of 45.2 and encapsulation efficiency of 88.8% and was chosen for further characterization and evaluation of its anti-bacterial and anti-biofilm activity.

#### Surface morphology of the chitosan-propolis nanoparticles by TEM

**Fig 3:**
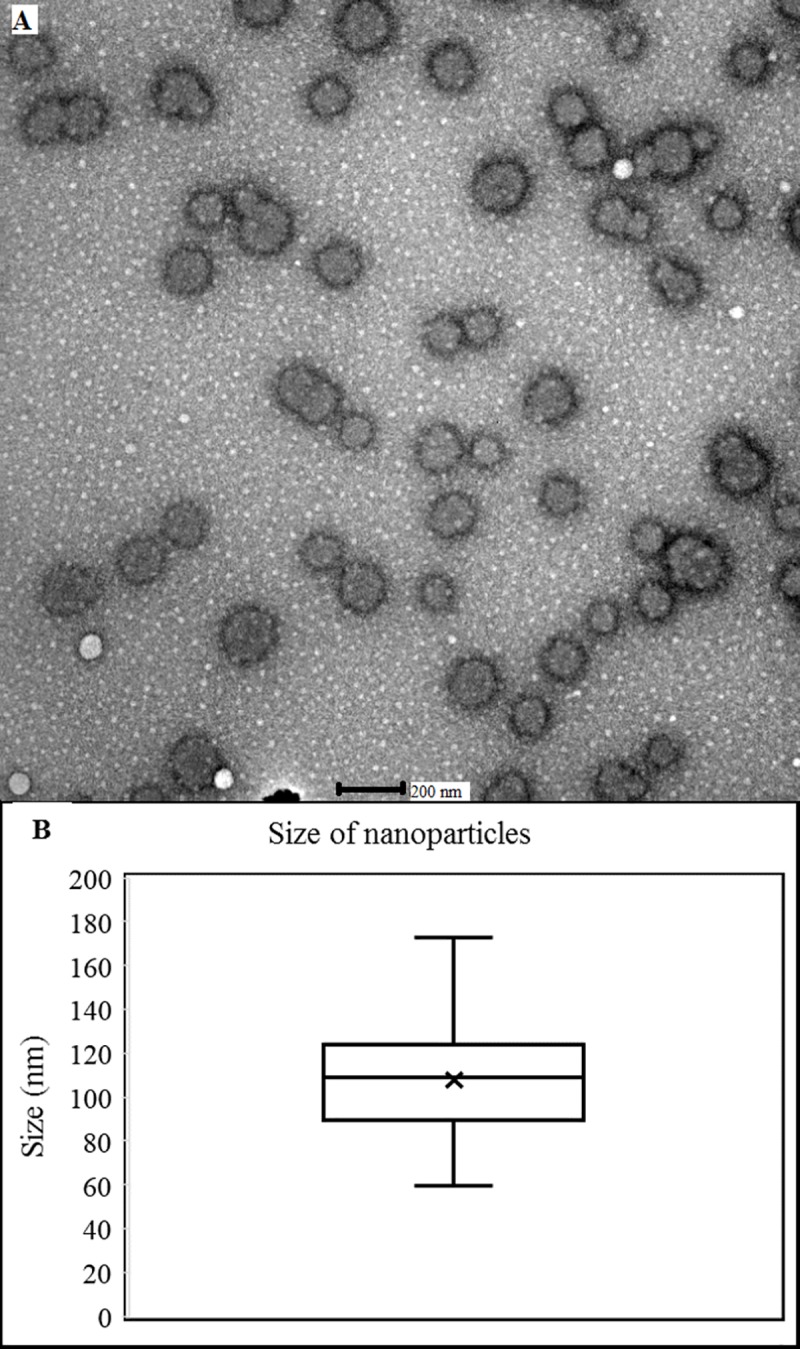
Transmission electron microscopy of chitosan-propolis nanoparticles. A) Chitosan-propolis nanoparticles from formulation F1 were stained with uranyl acetate and examined by transmission electron microscopy to determine the average particle size. B) Box plot depicting the average size of the nanoparticles calculated using ImageJ software.

The particle size and surface morphology of the chosen formulation F1 were further confirmed by transmission electron microscopy (TEM) (Fig 3). The nanoparticles were found to be spherical in shape with an average particle size of was 107.74 nm with a standard deviation of 24.76 nm. Although the average particle size measured by photon correlation spectroscopy is 247 nm, it is an estimation based on scattered light intensity [35]. The actual physical measurement is done by TEM. The chitosan-propolis nanoparticles were found to have a smooth surface with no surface drug crystals.

#### *In vitro* release of propolis from the nanoparticles

**Fig 4:**
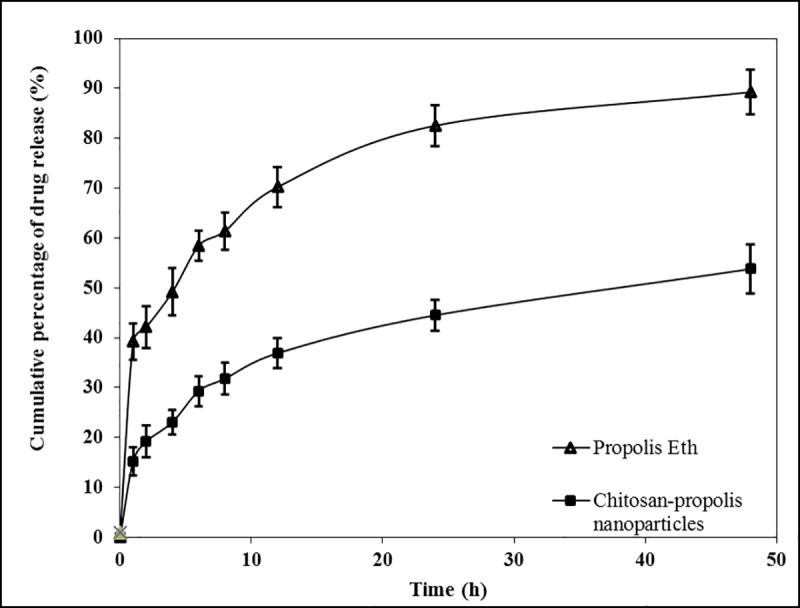
Rate of release of propolis from the formulation. Graph depicting the rate of release of propolis from the ethanol extract versus chitosan-propolis nanoparticles measured over 48 hours. Abbreviations: Propolis Eth: Propolis ethanol extract.

The rate and extent of release of a drug from a formulation depends on its morphology, size, nature of polymer, density of the particulate system and physicochemical properties of the drug [36]. Chitosan-propolis nanoparticles (formulation F1) had a burst release in the initial 2 hours followed by a steady, controlled and sustained release over 48 hours. At the end of 48 hours, the total release was only 53.8% (Fig 4). In contrast, the Eth and EA extracts of propolis exhibited a burst release of 39.2% within the first 2 hours and almost 90% of propolis was released within 48 hours. These results clearly indicate that formulation F1 released propolis in a sustained and controlled manner.

### Antibacterial efficacy of chitosan-propolis nanoparticles

#### Effect of nanoparticles on biofilm bacteria

**Fig 5:**
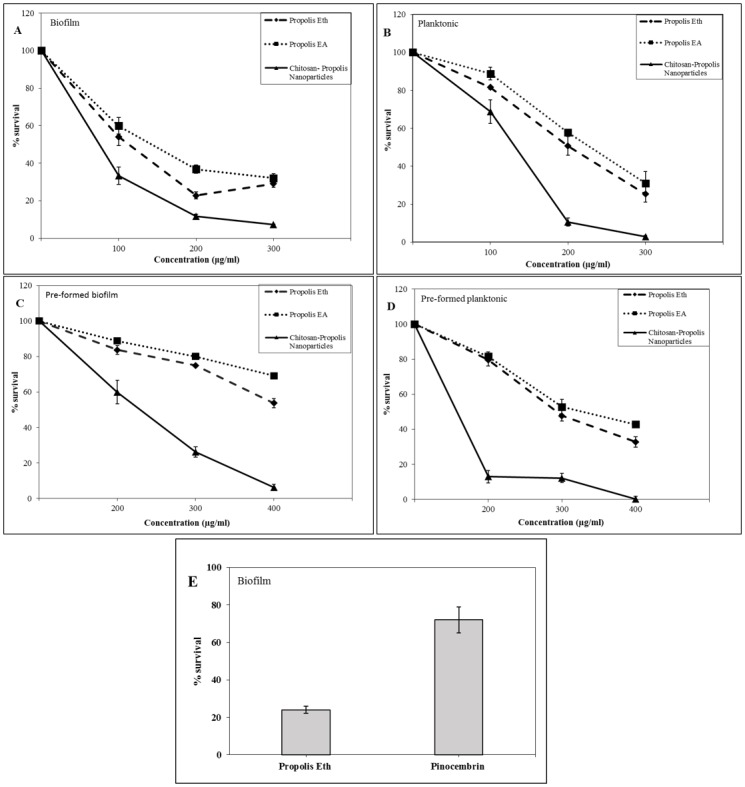
Percentage survival of bacteria in biofilm and planktonic form. Graphs describing the percentage survival of bacteria present in the biofilms (A and C) or in planktonic form (B and D) either co-treated (A and B) or pre-formed biofilm treated (C and D) with propolis Eth extract, EA extract or chitosan-propolis nanoparticle formulation F1. Graph depicting percentage survival of biofilm bacteria treated with pure pinocembrin or propolis Eth extract containing equivalent amount of pinocembrin (E).

The ability of formulation F1 in inhibiting biofilm formation by *E*. *faecalis* was evaluated by enumeration of the actual bacteria within the biofilm. Formulation F1 was found to inhibit *E*. *faecalis* biofilms better when compared to Eth and EA extracts of propolis (Fig 5). At a concentration of 100 μg/mL, treatment with nanoparticles resulted in ~30% survival of biofilm bacteria (Fig 5A). The effectiveness of chitosan-propolis nanoparticles increased with increase in concentration; at 300 μg/mL, only 10% of biofilm bacteria survived whereas about 30% survived in Eth or EA treatment. Planktonic bacteria were more sensitive to nanoparticles resulting in ~10% survival at 200 μg/mL (Fig 5B) concentration. At the same concentration, treatment with Eth and EA resulted in 50–60% survival of planktonic bacteria. Bacteria in biofilm as well as planktonic forms therefore showed more susceptibility to chitosan-propolis nanoparticles compared to Eth and EA extracts of propolis.

In order to verify if the biological activity of propolis can be attributed to the flavonoid pinocembrin, bacteria were allowed to form biofilm in the presence of 1 μg/mL of pinocembrin or 200 μg/mL of propolis Eth extract (equivalent to 1 μg/mL of pinocembrin). Pinocembrin caused 65% of biofilm bacteria to survive whereas Eth extract could reduce survival to about 25% (Fig 5E). The biological activity of pinocembrin is much lower than that of propolis Eth extract indicating that the anti-bacterial activity of propolis is not solely attributed to pinocembrin, but to a combined effect of different components.

#### Effect of nanoparticles on biofilm bacteria—Pre-formed biofilm

Pre-formed biofilm (16 hours) was more resistant to treatment with propolis extracts compared to the nanoparticles. When we used a concentration of 200 μg/mL, ~60% of biofilm bacteria survived when treated with chitosan-propolis nanoparticles whereas, ~90% of biofilm bacteria survived when treated with Eth and EA extracts of propolis (Fig 5C). At 300 μg/mL, the survival dropped to 20% when treated with nanoparticles while 80% survived with Eth and EA treatment. The planktonic bacteria from pre-formed biofilms also displayed high susceptibility to nanoparticles (~10% survival at 200 μg/mL) compared to Eth and EA treatment (~80% at 200 μg/mL) (Fig 5D). This clearly demonstrates that nanoparticles are capable of penetrating pre-formed biofilm and eradicate bacteria. This can be attributed to their enhanced penetration ability due particle size.

#### Visualization of biofilm

**Fig 6:**
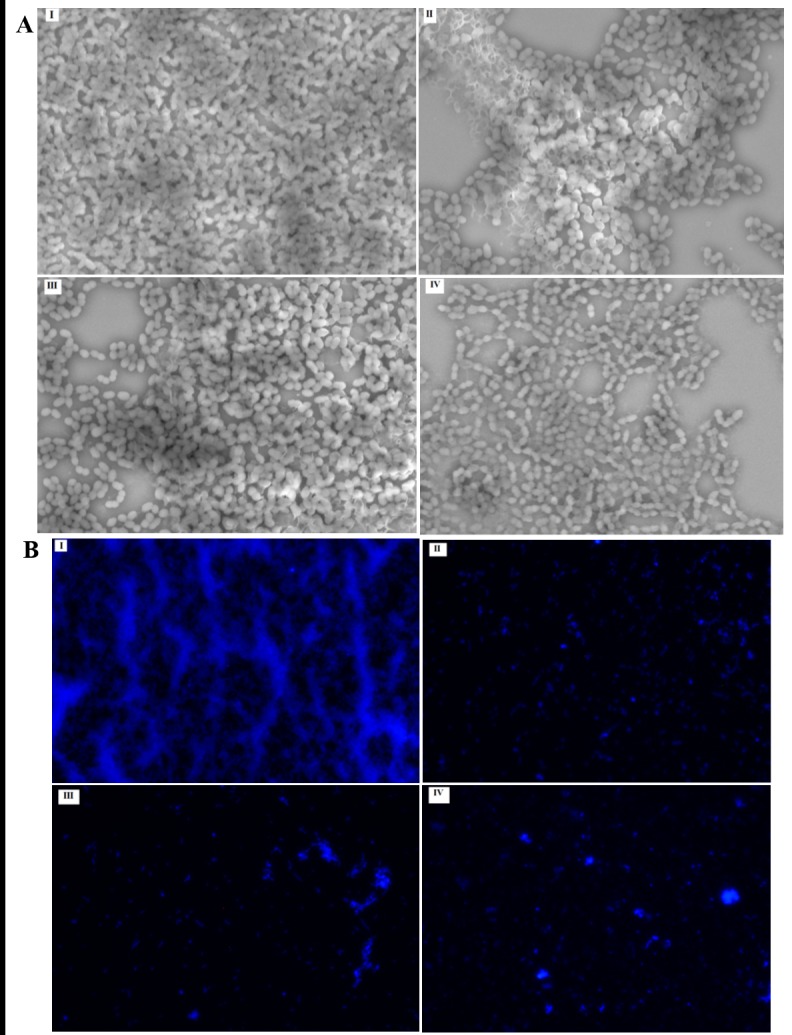
Scanning electron microscopy and calcofluor white fluorescent microscopy of bacterial biofilms. (A) Scanning electron microscopy picture depicting biofilm bacteria and (B) fluorescence microscopy picture depicting biofilm formation by (I) untreated control bacteria, (II) bacteria treated with Eth or (III) EA extracts of Malaysian propolis or (IV) chitosan-propolis nanoparticles.

Scanning electron microscope (SEM) was used to observe the bacteria in the biofilms (Fig 6A) whereas, visualization of biofilm matrix was carried out by calcofluor white staining followed by imaging using a fluorescent microscope (Fig 6B). When observed using a SEM, a thick layer of well-formed bacterial biofilm was seen in the control group (Fig 6A-I) whereas, in the presence of nanoformulation F1, disrupted biofilm was observed with intermittent patches of clear areas depicting the inhibition of biofilm (Fig 6A-IV). Treatment with Eth and EA showed partial disruption of the biofilm (Fig 6A-II and 6A-III). This again indicates that nanoformulation F1 is more effective in disrupting bacterial biofilm.

Under fluorescent microscope, a thick and intact layer of biofilm was seen in the control group (Fig 6B-I) whereas, in the groups treated with Eth or EA or chitosan-propolis nanoformulation F1 (Fig 6B-II, 6B-III and 6B-IV), the biofilm layer was thin or discontinuous depicting structural deformity indicating disruption of biofilm mediated by propolis treatment. This figure only indicates staining of biofilm matrix but not bacterial numbers. All three treatments are capable of interfering with biofilm matrix formation.

#### Effect of chitosan-propolis nanoformulation on bacterial gene expression

Bacteria living in the biofilm have a different microenvironment when compared to their planktonic counterparts and hence the former alter their gene signature to facilitate biofilm formation, which could in turn increase their survival and offer resistance to penetration by antibacterial agents. Modulation of gene expression of cytolysin genes and virulence genes in *E*. *faecalis* biofilm bacteria was analyzed by real-time quantitative PCR. Gene expression level in planktonic bacteria was used to normalize the same in biofilm bacteria. Increase in gene expression in biofilm bacteria by (>1.5 fold) was considered significant.

**Fig 7:**
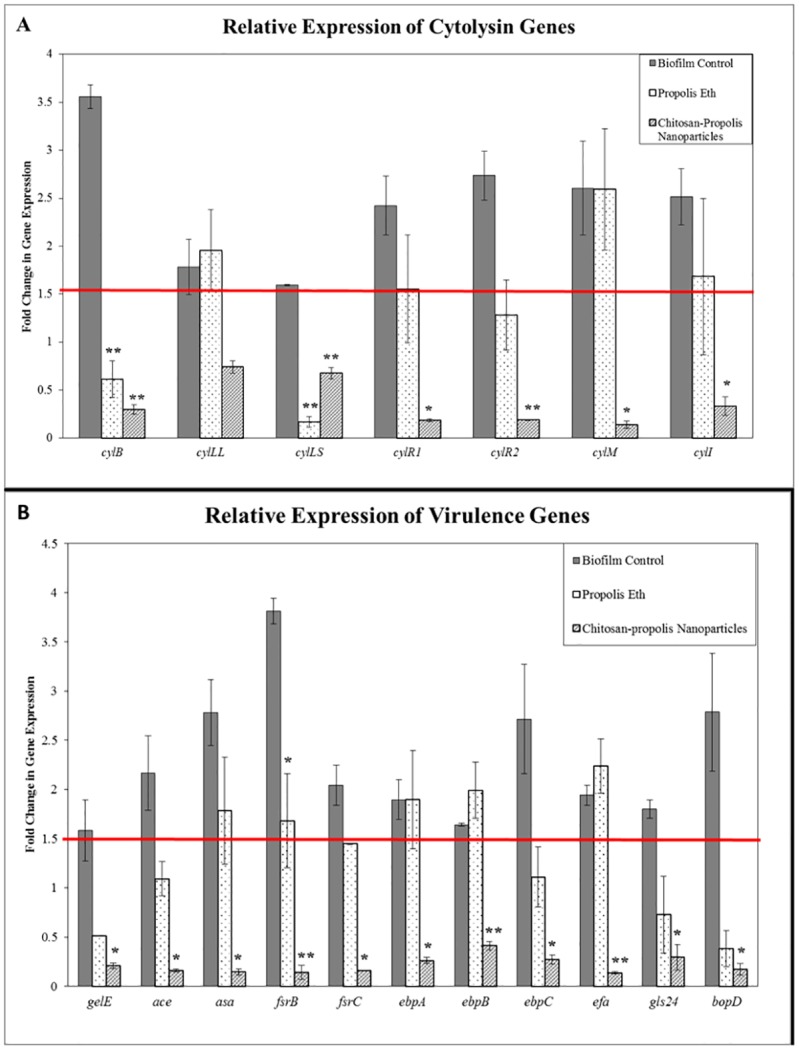
Quantitative PCR analysis of gene expression in *E*. *faecalis*. Relative expression of genes regulating cytolysin genes (A) or virulence genes (B) in *E*. *faecalis* determined by quantitative real-time PCR analysis. Total RNA was extracted from bacteria in different treatment groups (biofilm control, biofilm treated with propolis Eth extract and biofilm treated with nanoparticles), converted to cDNA and analyzed by qPCR with the respective primers. Abbreviations: Propolis Eth: Propolis ethanol extract.

The genes that were upregulated were cytolysin producing genes (*cylB*, *cylL*_*L*_, *cylL*_*S*_, *cylM* and *cyl1*) and cytolysin regulatory genes (*cylR1* and *cylR2*), which are involved in the invasive mechanisms. The other set of genes upregulated are associated with biofilm virulence factors (*gelE*, *ace*, *asa*, *fsrB*, *fsrC*, *ebpA*, *ebpB*, *ebpC*, *efa*, *gls24* and *bopD*), which will ensure the survival by attachment and colonization (Fig 7). Treatment of biofilms with Eth extract of propolis reduced the expression of only a few genes but most virulence and invasive gene expressions were not significantly decreased. Treatment with chitosan-propolis nanoformulation on the other hand, was able to significantly decrease the expression of most of the genes studied. Down regulation of cytolysin and biofilm-forming genes after treatment with nanoparticles proves that the nanoparticles are able to modulate the expression of biofilm-associated genes, thereby rendering the bacteria susceptible to treatment. This demonstrates that chitosan-propolis nanoformulation is an ideal anti-biofilm agent that not only penetrates biofilms but also renders the bacteria susceptible to subsequent treatment with antibacterial agents [37, 38].

Taken together, our studies have clearly demonstrated the role of Malaysian propolis in combating biofilms formed by *E*. *faecalis*. Treatment of biofilms with chitosan-propolis nanoparticles enhances the treatment efficacy, biofilm-penetrating ability as well as the susceptibility of bacteria to antibacterial agents.

## Discussion

This is the first report on the characterization of Malaysian propolis and evaluation of its anti-bacterial effect against *E*. *faecalis* biofilms. Brazilian propolis has been best characterized and 13 different types of propolis have been documented [39, 40]. The composition of Malaysian propolis was analyzed by extraction with two organic solvents—ethanol and ethyl acetate followed by HPLC analysis to identify the constituents. Ethanol is a widely used solvent for extracting biologically active compounds from propolis [41]. A previous study comparing the extraction methods used for propolis had concluded that ethanol extracts have the maximum anti-bacterial effect, which can be attributed to their highest content of phenolics and flavonoids [42], which validates our findings that Eth extract is more effective than EA extract in terms of anti-bacterial effect.

### Pinocembrin and other flavonoids in propolis

Flavonoids are the major constituents of propolis and therefore majorly contribute to its therapeutic actions [28]. Propolis from Chile has been reported to contain nine flavones/flavonol derivatives, one flavanone, eight dihydroflavonols and nine phenyl-propanoids [43]. Cinnamic acid derivatives and flavonoids are capable of affecting bacterial membrane potential by interfering with ion permeability leading to inhibition of bacterial mobility [44]. The chemical components commonly present in propolis are pinocembrin, caffeic acid, cinnamic acid, coumaric acid, caffeic acid, ferulic acid, artepillin C, chrysin, galangin, kaempferol and quercetin [45]. The composition differs with geographic location, botanical origin, season and time of collection [28–30].

Pinocembrin is a dihydroxy flavanone that contributes to the anti-bacterial effect of propolis along with other components. Argentinean propolis has been reported to have a high content of pinocembrin [46]. Pinocembrin has been reported to demonstrate strong antimicrobial activity against *E*. *faecalis*, *Staphylococcus epidermidis*, *S*. *aureus* (methicillin and gentamycin resistant), *Bacillus cereus*, *Escherichia coli*, *Klebsiella pneumonia*, *Pseudomonas aeruginosa*, *Cryptococcus neoformans* and *Candida albicans* [47, 48] and is also reported to inhibit both Gram-positive and Gram-negative bacteria and induce bacterial cell lysis [49].

Pinocembrin content is almost similar (about 4%) in propolis extracted from different geographical locations across the world [50]. Pinocembrin has been identified as the active component of United State propolis and it is capable of disrupting acryl-homoserine lactone-dependent quorum sensing in bacteria [51]. The anti-bacterial effect of Malaysian propolis is attributed to the flavonoids/flavones present, not limited to the identified compounds pinocembrin and kaempferol.

### Nanoparticles facilitate effective eradication of biofilm

Nanoparticle-based drug delivery systems offer enhanced drug stability, treatment efficacy and penetration power compared to a pure drug solution [52, 53]. Drugs encapsulated in nanostructured carriers with the right size and surface charges are resistant to enzymatic degradation [54]. The efficacy of nanoparticles, especially metal-based nanoparticles against *E*. *faecalis* biofilms has been well documented [55, 56]. Although most nanoparticle systems used for treating biofilms contain metals or drugs, nanoformulations with natural products offer broader potential for therapy. Propolis is a natural product with antibacterial property and is ideal to be developed as nanoparticles.

Though ethanol and ethyl acetate extracts of propolis have anti-bacterial effect, due to their poor solubility in aqueous solutions, their concentration in a solution could not be increased beyond an optimal range. When prepared as nanoparticles, they are dispersible in water and are effective in reducing bacterial population by ~90%. The nanoparticles, with their enhanced solubility, bioavailability, and efficacy probably facilitate higher cellular uptake of propolis endowing higher penetration power.

Although chitosan-propolis nanoparticles could reduce bacterial numbers in biofilms by ~70% (at 200 μg/ml) upon co-treatment, only 40% bacteria in pre-formed biofilms could be eradicated at the same concentration. Treatment of pre-formed biofilm with higher concentration of nanoparticles (300 μg/ml) could eradicate ~75% of bacteria within the biofilm. Once biofilm matrix with extra polymeric substances is in place, impermeable property inhibits the penetration of therapeutic agents. Nanoparticles are able to penetrate the biofilm owing to their nano-scale particle size [57, 58].

A mixture of virulence factors contribute to the pathogenesis of *E*. *faecalis* infections, many of them are involved in bacterial adhesion to host cells or abiotic surfaces leading to biofilm formation. Some are involved in resistance to anti-microbial agents. The gene products of *asa* and *cyl* are involved in aggregation of bacteria [59]. *Efa* contributes to adhesion [60] and *gelE* encodes for gelatinase that is capable of hydrolysing biological peptides [61]. *cyl* is a bacteriocin that is capable of lysing prokaryotic as well as eukaryotic target cells. It is particularly effective against Gram-positive bacteria [62]. Many studies with clinical strains of *E*. *faecalis* have reported the expression of *efa*, *gelE*, *asa*, *cyl* in the isolates [63, 64]. Genes involved in adhesion and antibiotic resistances were found to be overexpressed in biofilm bacteria [65] as observed in our system. Treatment with anti-bacterial agents is reported to be capable of reducing virulence gene expression [66] corroborating our findings.

A previous study comparing minimum bactericidal concentration of planktonic and biofilm bacteria had concluded that biofilm bacteria demonstrated greater extent of tolerance to antibiotics compared to planktonic bacteria [67]. *E*. *faecalis* exhibits a high level of resistance to commonly used antibiotics like tetracycline, methicillin and vancomycin [68]. Nanoparticles offer huge potential against multi-drug resistant bacteria, and serve as alternatives to antibiotics and conventional antimicrobial agents [69]. Our findings using chitosan-propolis nanoparticles is suggestive of both antimicrobial and anti-biofilm agent which can have implications in circumstances where it needs removal of bacteria as well as biofilms, which we encounter in many clinical scenario like wounds and postoperative lesions.

In dentistry, *E*. *faecalis* is known to form oral biofilms, is often associated with endodontic failure and is found viable in the filled root canals even one year after surgery [70]. Topical application of antibiotics or chlorhexidine is ineffective in eradication of such biofilms. Similarly, biofilms formed in implants and catheters necessitate their replacement rather than eradication of the biofilms. Development of a naturally occurring product, chitosan bound propolis against biofilm bacteria offers immense potential for developing chitosan-propolis nanoparticles as topical sterilizing agents.

## Conclusion

*E*. *faecalis* is reported to be resistant to most commercial antibiotics, forms biofilms, which protect the bacteria within and inhibit infiltration by antimicrobial agents. The chitosan-propolis nanoformulation (F1) developed had ideal physicochemical parameters and inhibited bacterial growth as well as biofilm formation by *E*. *faecalis*. It altered the expression of genes responsible for virulence and biofilm formation, causing the bacteria to lose their characteristic features that enable biofilm formation thereby rendering them susceptible to treatment. This nanoformulation therefore has the potential to be developed as therapeutic agent combating bacterial biofilms.

## Supporting information

S1 Fig Comparative effect of Eth and EA extracts of Malaysian propolis on biofilm formation. Graph depicting the percentage reduction in biofilm mass treated with ethanol or ethyl acetate extracts of Malaysian propolis. *E*. *faecalis* was cultured in 96-well plates and allowed to form biofilms in the presence or absence of Eth or EA extracts of Malaysian propolis. After 24 h, the plates were washed, stained with crystal violet and the amount of crystal violet released from the biofilm was estimated. Abbreviations: Propolis EA: Propolis ethyl acetate extract; Propolis Eth: Propolis ethanol extract.
(DOCX)

S1 Table Primer sequences used for quantitative PCR. (DOCX)

## Abbreviations

*E. faecalis*: *Enterococcus faecalis*
EA: Ethyl acetate
Eth: Ethanol
HPLC: High performance liquid chromatography
SEM: Scanning electron microscope
