# Correction: Chitosan-propolis nanoparticle formulation demonstrates anti-bacterial activity against *Enterococcus faecalis* biofilms

**DOI:** 10.1371/journal.pone.0176629

**Published:** 2017-04-20

**Authors:** Teik Hwa Ong, Ebenezer Chitra, Srinivasan Ramamurthy, Rajinikanth Paruvathanahalli Siddalingam, Kah Hay Yuen, Stephen Periathamby Ambu, Fabian Davamani

There are errors in the author affiliations. The affiliations should appear as shown here: Teik Hwa Ong^1^, Ebenezer Chitra^2^, Srinivasan Ramamurthy^3^, Rajinikanth Paruvathanahalli Siddalingam^3^*, Kah Hay Yuen^4^, Stephen Periathamby Ambu^1^, Fabian Davamani^2^

**1** School of Postgraduate Studies, International Medical University, Bukit Jalil, Kuala Lumpur, Malaysia, **2** Division of Applied Biomedical Science and Biotechnology, School of Health Sciences, International Medical University, Bukit Jalil, Kuala Lumpur, Malaysia, **3** School of Pharmacy, International Medical University, Bukit Jalil, Kuala Lumpur, Malaysia, **4** School of Pharmaceutical Sciences, Universiti Sains Malaysia, Gelugor, Penang, Malaysia.

*Current address: School of Pharmacy, Taylors University, Selangor, Malaysia
