# Supplementary material for: Chitosan-propolis nanoparticle formulation demonstrates anti-bacterial activity against Enterococcus faecalis biofilms
Source: PLoS One. 2017 Mar 31;12(3):e0174888. doi: 10.1371/journal.pone.0174888 (PMC5376299; doi:10.1371/journal.pone.0174888)
Supplement: S1 Fig — Graph depicting the percentage reduction in biofilm mass treated with ethanol or ethyl acetate extracts of Malaysian propolis. E. faecalis was cultured in 96-well plates and allowed to form biofilms in the presence or absence of Eth or EA extracts of Malaysian propolis. After 24 h, the plates were washed, stained with crystal violet and the amount of crystal violet released from the biofilm was estimated. Abbreviations: Propolis EA: Propolis ethyl acetate extract; Propolis Eth: Propolis ethanol extract. (DOCX) [file pone.0174888.s001.docx]

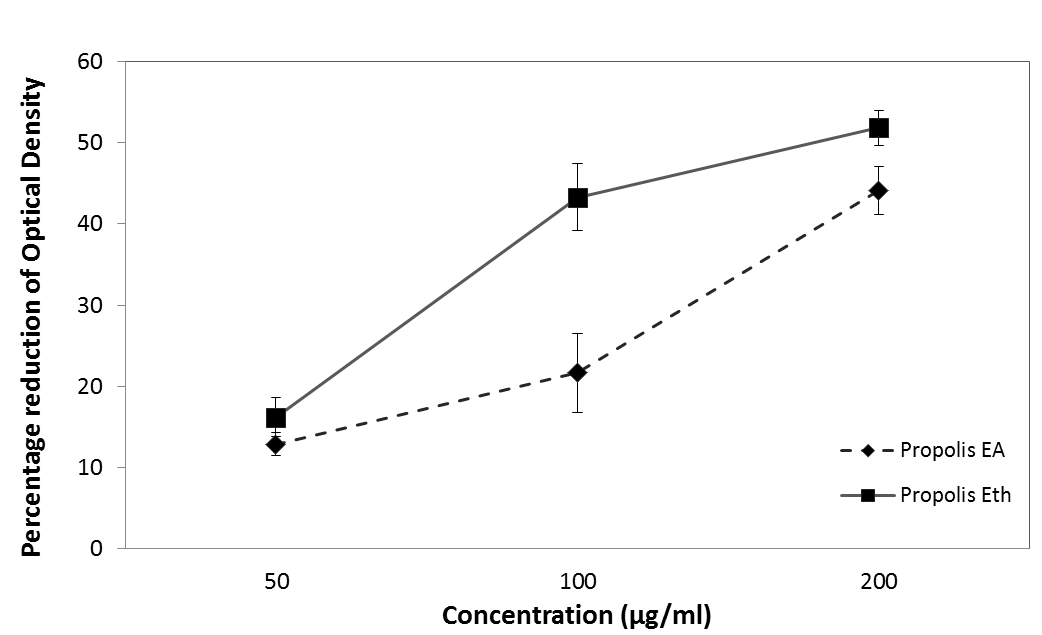


**S1 Fig. Comparative effect of Eth and EA extracts of Malaysian propolis on biofilm formation**

Graph depicting the percentage reduction in biofilm mass treated with ethanol or ethyl acetate extracts of Malaysian propolis. *E. faecalis* was cultured in 96-well plates and allowed to form biofilms in the presence or absence of Eth or EA extracts of Malaysian propolis. After 24 h, the plates were washed, stained with crystal violet and the amount of crystal violet released from the biofilm was estimated.

Abbreviations: Propolis EA, Propolis ethyl acetate extract; Propolis Eth, Propolis ethanol extract
