# Supplementary material for: Chitosan-propolis nanoparticle formulation demonstrates anti-bacterial activity against Enterococcus faecalis biofilms
Source: PLoS One. 2017 Mar 31;12(3):e0174888. doi: 10.1371/journal.pone.0174888 (PMC5376299; doi:10.1371/journal.pone.0174888)
Supplement: S1 Table — (DOCX) [file pone.0174888.s002.docx]

**S1. Primer sequences used for quantitative PCR**

| **Gene** | **Primer** | **Primer Temperature (ºC)** | **Gene Function** |
| --- | --- | --- | --- |
| *gls24^1^* | F: taacagtcgatggcggcttt  R: cagcgacttgttttttaccaacttc | 55 | General stress protein |
| *bopD^2^* | F: acggcacggaatttgggtaaac  R: ggcttcctcgttgatggcttc | 55 | Biofilm formation |
| *gelE^1^* | F: cggaacatactgccggtttaga  R: tggattagatgcacccgaaat | 55 | Gelatinase |
| *ace^1^* | F: cggcgactcaacgtttgac  R: tccagccaaatcgcctactt | 55 | Collagen-binding adhesin |
| *asa^1^* | F: gatacaaagccaatgtcgttcct  R: taaagagtcgccacgtttcaca | 53 | Aggregation substance |
| *fsrB^1^* | F: tgctcaaaaagcaaagccttataa  R: gatgacgagaccgtagagtattactgaa | 45 | Efae regulator |
| *fsrC^1^* | F: gcttatttggaagaacaacgtatcaa  R: cgaaacatcgctagctcttcgt | 55 | Efae regulator |
| *ebpA^3^* | F: caacaacaccagggctttttg  R: accggaccagtcaacgactaag | 53 | Biofilm-associated pili |
| *ebpB^3^* | F: cgtacaggcggcaagtcttt  R: aggtattcccccgcttgattt | 56 | Biofilm-associated pili |
| *ebpC^3^* | F: gcggcacactaaaattcgttta  R: gtcgtcggtatgaccgttatca | 55 | Biofilm-associated pili |
| *efa^1^* | F: tgggacagaccctcacgaata  R: cgcctgtttctaagttcaagcc | 53 | Enterococcal surface protein |
| *cylB^1^* | F: gaaaagattgaagtacgttgcg  R: ttctactagtgtactttgattaccataataatt | 55 | cytolysin toxin |
| *cylL_L_^1^* | F: ctgttgcggcgacagct  R: ccaccaacccagccacaa | 55 | cytolysin toxin |
| *cylL_S_^1^* | F: gctaaataaggaaaatcaagaaaactattactc  R: caaaagaaggaccaacaagttctaatt | 55 | cytolysin toxin |
| *cylR1^1^* | F: tttatttttttattggatatcatttctgtagtc  R: ttcgctcatctttttttgaatcag | 55 | cytolysin regulatory |
| *cylR2^1^* | F: ccaaagtgaattagctgctttattagaa  R: ttaatgctaactgtaaagaagggttatatttatt | 55 | cytolysin regulatory |
| *cylM^1^* | F: tcggacacggtatatatagctatgt  R: ttctactagtgtactttgattaccataataatt | 55 | cytolysin toxin |
| *cyl1^1^* | F: acttctcgtaatctttactcttgtttttg  R: ctaagtgccctaactcatgtacaa | 55 | cytolysin toxin |
| *23sRNA*^1^ | F: cctatcggcctcggcttag  R: agcgaaagacaggtgagaatcc | 57 | Internal control |

**References:**

1. Shepard BD, Gilmore MS. Differential Expression of Virulence-Related Genes in *Enterococcus faecalis* in Response to Biological Cues in Serum and Urine. *Infection and Immunity*. 2002;70(8):4344-4352.

2. Hufnagel M, Koch S, Creti R, Baldassarri L, Huebner J. A putative sugar-binding transcriptional regulator in a novel gene locus in *Enterococcus faecalis* contributes to production of biofilm and prolonged bacteremia in mice. *J Infect Dis*. 2004;189(3):420-430.

3. Gao P, Pinkston KL, Nallapareddy SR, van Hoof A, Murray BE, Harvey BR. *Enterococcus faecalis rnjB* is required for pilin gene expression and biofilm formation. *J Bacteriol*. 2010;192(20):5489-5498.
